# Development and testing of a new system for assessing wheel-running behaviour in rodents

**DOI:** 10.1186/s13104-016-2059-6

**Published:** 2016-05-05

**Authors:** Taylor Chomiak, Edward W. Block, Andrew R. Brown, G. Campbell Teskey, Bin Hu

**Affiliations:** Division of Experimental Neuroscience, Department of Clinical Neurosciences, Hotchkiss Brain Institute, Cumming School of Medicine, University of Calgary, 3330, Hospital Drive NW, Calgary, AB T2N 4N1 Canada; Department of Cell Biology and Anatomy, University of Calgary, Calgary, AB T2N 4N1 Canada

**Keywords:** Wheel, Running, Rodent, Motor, Skill, Procedural, Learning

## Abstract

**Background:**

Wheel running is one of the most widely studied behaviours in laboratory rodents. As a result, improved approaches for the objective monitoring and gathering of more detailed information is increasingly becoming important for evaluating rodent wheel-running behaviour. Here our aim was to develop a new quantitative wheel-running system that can be used for most typical wheel-running experimental protocols.

**Findings:**

Here we devise a system that can provide a continuous waveform amenable to real-time integration with a high-speed video ideal for wheel-running experimental protocols. While quantification of wheel running behaviour has typically focused on the number of revolutions per unit time as an end point measure, the approach described here allows for more detailed information like wheel rotation fluidity, directionality, instantaneous velocity, and acceleration, in addition to total number of rotations, and the temporal pattern of wheel-running behaviour to be derived from a single trace. We further tested this system with a running-wheel behavioural paradigm that can be used for investigating the neuronal mechanisms of procedural learning and postural stability, and discuss other potentially useful applications.

**Conclusions:**

This system and its ability to evaluate multiple wheel-running parameters may become a useful tool for screening new potentially important therapeutic compounds related to many neurological conditions.

**Electronic supplementary material:**

The online version of this article (doi:10.1186/s13104-016-2059-6) contains supplementary material, which is available to authorized users.

## Findings

Wheel running is one of the most widely studied behaviours in laboratory rodents. For example, it is used to evaluate motor deficits in rodent models of Multiple Sclerosis and Parkinson’s Disease, and hyperactivity in a genetic model of attention-deficit-hyperactivity-disorder [1–5]. It has also been used to study exercise in rehabilitation after spinal cord injury and for autistic-like behavioural phenotyping [6, 7]. In addition, the use of voluntary wheel running is important for studying motivation and modelling instrumental goal-directed behaviour in rodents [8, 9]. Here, instrumental behaviour (running) is required to turn the wheel which then provides positive sensorimotor-feedback to reinforce goal-directed behaviour that is highly motivational [8, 9]. A novel running-wheel task has also been developed to model and investigate neural mechanisms of motor-skill procedural learning and memory [10], an area of research that can offer important contributions in the understanding of both motor function and learning and memory [10, 11].

Quantification of wheel running behaviour has typically focused on the number of revolutions per unit time as an end point measure [10, 12–17]. For this, the simplest approach is to use a mechanical counter to count the absolute number to revolutions [10, 13]. Of course, one problem with this is that if it is not automated, the logging of information can often be impractical when more time points are required for higher temporal resolution and/or when longer monitoring periods are needed. Furthermore, most currently utilized experimental set-ups are unable to establish and record wheel-running direction. Knowing when and what direction the rodent is running is, for example, extremely important when investigating mechanisms of direction-dependent neural activity [18]. Previous studies have utilized automated systems to report more detailed information, like maximum velocity (revolutions per min), using a rotation sensor with 16/turn or a photocell counter [1, 16, 17]. However, although the temporal resolution is improved, these automated approaches still lack directionality, and thus the ability to evaluate relative differences in continuous wheel rotation fluidity. Hence, as a better approach to wheel running quantification and more objective monitoring is increasingly becoming important, we have devised a system that can provide a continuous waveform amenable to real-time integration with high-speed video if desired that is ideal for most typical wheel-running experimental protocols. We further test this system with a running-wheel behavioural paradigm that can be used for investigating the neuronal mechanisms of procedural learning and discuss other potentially useful applications.

### System design and utility

**Fig. 1:**
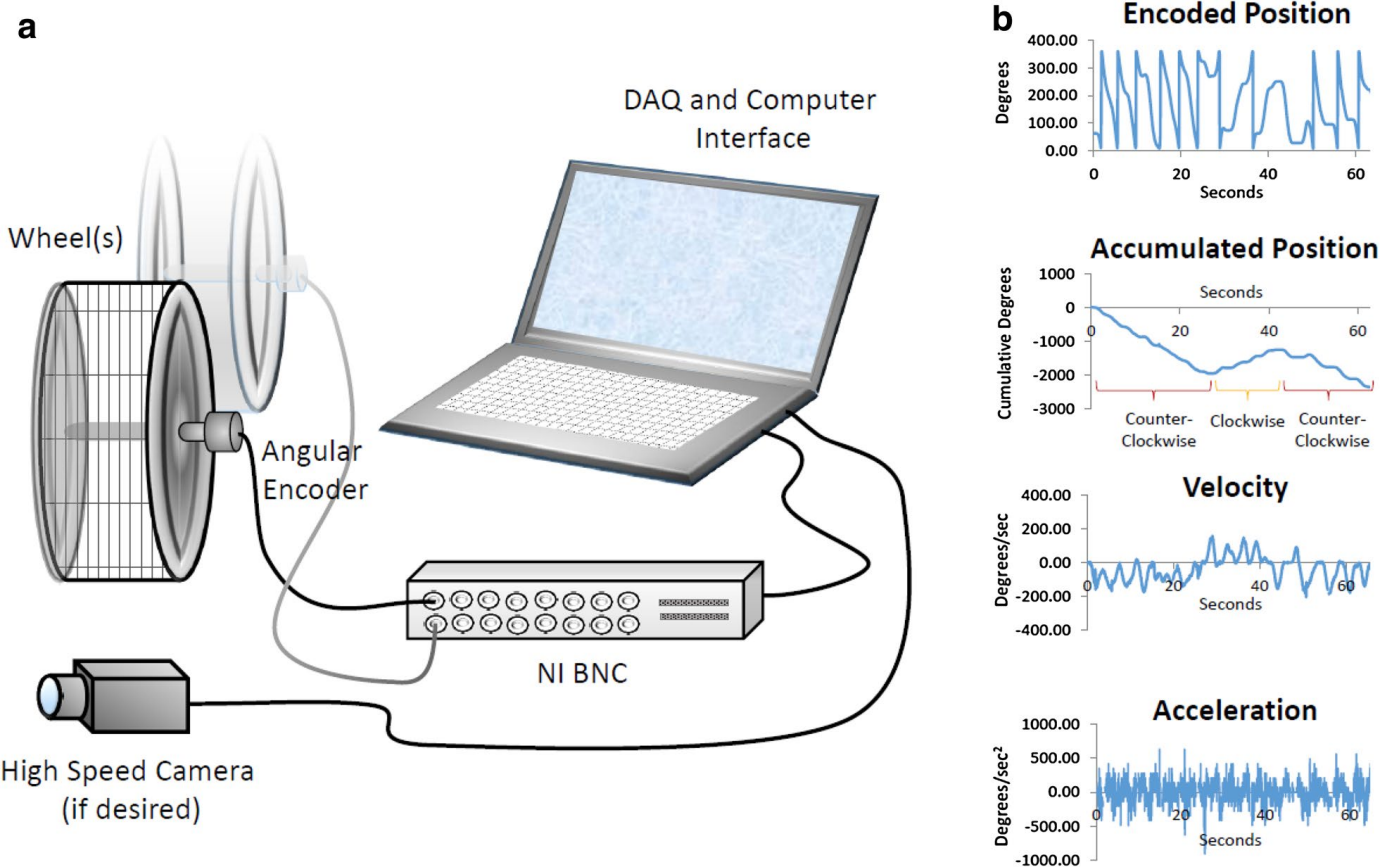
A schematic overview of the system used for assessing wheel-running behaviour in rodents. **a** A schematic overview of the system set-up. The system using LabVIEW system design software (National Instruments). A laptop computer (IBM ThinkPad) with a National Instruments A/D board (NI DAC-Card 6024E, 200 kSamples/s, 16 channels), a breakout box (National Instruments BNC), and a high speed IEEE 1394a port was used to run in-house software to collect raw position data (see **b**). Additional details are shown in Additional file 1: Figure S1. **b** Examples of wheel-running data. Cumulative position (or distance), velocity, and acceleration can all be derived from the raw position trace (*top* to *bottom*). The velocity trace represents d*x*/d*t*, and the acceleration trace represents d*v*/d*t*. Direction (i.e. clockwise or counter-clockwise) is noted in the accumulated position trace and reflected as “+” and “−” velocities in the velocity trace. See text for details

We first designed a system for detailed data collection (Fig. 1a; Additional file 1: Figure S1). This consisted of a laptop computer (Lenovo ThinkPad W500) with a National Instruments A/D PCMCIA card (NI DAC-Card 6024E, 200 kSamples/s, 16 channels), a breakout box (National Instruments BNC-2090), and a high speed IEEE 1394a port, as well as an angular encoder (model: 6639S-1-103; Digi-Key, Thief River Falls, MN, USA) mounted on the wheel axis to record angular position of the wheel at a sampling rate of 20 Hz. The encoder outputs 0–5 V, corresponding to 0°–360° and then wraps back to 0° upon a complete rotation. This can also be plotted as cumulative or accumulated position for total degrees (Fig. 1b), or converted to cumulative distance if desired [i.e. every 360° = 1.13 m (2πr)]. The position traces can also be used to evaluate total degrees (or distance) in either clockwise or counter-clockwise directions (Fig. 1b). Mathematical differentiation (d*x*/d*t*) on the position trace yields instantaneous velocity (Fig. 1b), and a second differentiation (d*v*/d*t*) yields instantaneous acceleration (Fig. 1b). Due to a “break-then-make” connection, the encoder output approaching the transition points (i.e. 360°→0° or 0°→360°) results in a “jump” that can been seen on the raw encoded position trace as a vertical line (i.e. the saw tooth pattern). These points can be left in and used as a fixed position point and marker of individual rotations in the velocity trace (see velocity trace in Additional file 1: Figure S1 for example), or they can be filtered out by removing three to six data points (0.15–0.3 s) on each side in the trace (Fig. 1b). The raw waveform can also be coupled to a high-speed camera (Basler A601f, 640 × 480 pixels, 100 frames/s) with the angular position attached to each video frame (Additional file 2: Video S1).

**Fig. 2:**
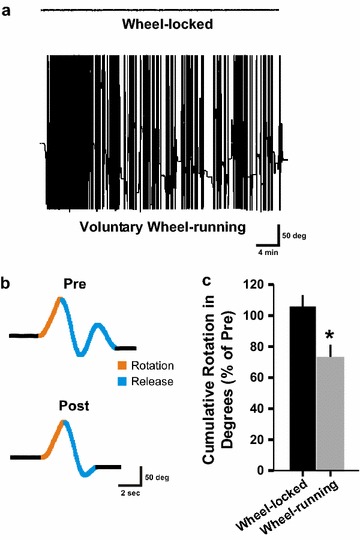
Using the system for wheel-skill learning for evaluating procedural motor learning. **a** Between wheel-skill evaluation (i.e. between the pre-test and post-test), the training period was monitored continuously for the wheel-locked group (*upper trace*) and for the wheel-running group (*lower trace*). The running-wheel group had free access to voluntary wheel running, while the wheel was locked in the locked-wheel group (~40 min session). The wheel running group ran an average of 404 ± 40 m over this period. *Vertical* changes in the *trace* with time represent rotations of the wheel (i.e. Δ_position_ ≠ 0°) while a *horizontal trace* represents no rotation (i.e. Δ_position_ = 0°). **b** Clear examples of a pre (*top*) and post (*bottom*) trace from the same wheel-running rat showing the rotation phase (*orange*) and the release phase (*blue*) of wheel-skill learning. **c** Summarized wheel-skill learning data for a single session for the wheel-locked group (*black*) and the wheel-running group (*grey*). *Asterisk* denotes p < 0.05

We next wanted to test our system. To this end, we evaluated wheel-skill learning, a reliable evaluation of procedural motor learning and postural stability (see “Methods” section and [10]). Rats were first randomly assigned to one of two groups; the wheel-running group or the locked-wheel group. The only difference between the two groups is that rats in the wheel-running group were permitted to freely practice wheel running between the pre and post tests. As might be expected with the option to run, animals in the wheel-running group did indeed run more than that of the wheel-locked group as shown in Fig. 2a. Our results validate previous results [10] that wheel-running experience during a single 40 min session is associated with significant improvements in wheel-skill learning (Fig. 2b; t_(6)_ = 2.805, p < 0.05). Together, our system offers an objective and efficient method for evaluating and quantifying wheel rotation.

**Fig. 3:**
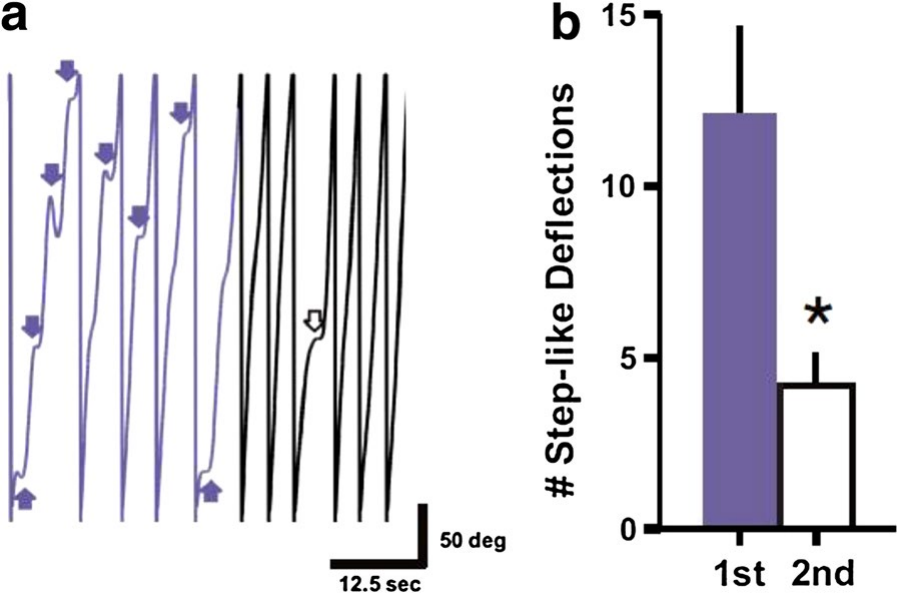
Using the system to evaluate wheel rotation fluidity. **a** As noted previously in video recordings [10], during the beginning of the running-wheel training (i.e. when naive rats are first exposed to the running wheel), rats are unable to run with an appropriate speed to remain at the bottom of the wheel. As a result, the rat often moves too fast or too slow relative to the speed of the wheel, thus resulting in poor wheel rotation fluidity (i.e. “step-like deflections” indicated by *arrows* in the *trace*). However, rats rapidly learn to remain in the bottom while running. **b** Summarized step-like deflection data for 5 initial rotations (*purple*) and 5 subsequent rotations (*white*) illustrating that despite the same number of rotations, there is clear quantitative differences of how these 5 revolutions are performed. *Asterisk* denotes p < 0.05

While our system is ideal for quantifying striatal-dependent procedural learning, it has several other potentially useful applications, three of which are highlighted here. First, the fact that our system incorporates wheel rotational direction will allow for the evaluation of relative differences in continuous wheel-rotation fluidity (Figs. 1, 3a). This is important as although end point measures may be the same (e.g. number of revolutions), how a subject accomplishes the task may not be (Fig. 3b; t_(3)_ = 3.52, p < 0.05). Indeed, in rodent models of Parkinson’s disease for example, compensational strategies have been observed following lesions to the motor system [19]. Thus, being able to monitor how an animal performs that task can be equally if not more important than simply evaluating whether the animal completes the task. In fact, by accounting for wheel-rotation fluidity, our system, unlike other wheel-running experimental set-ups, may also be useful in evaluating features of how different animal models of disease accomplish the task of wheel running. Accordingly, this system and its ability to evaluate multiple wheel-running parameters may become a useful tool for screening new potentially important therapeutic compounds.

Second, previous studies of mice selectively bred for high voluntary wheel running have suggested that the hyperactivity is associated with dysfunction in the dopaminergic neuromodulatory system and that high-running mice may represent a useful genetic model for attention-deficit-hyperactivity-disorder [2]. However, unlike that of Rhodes and Garland [2] that calculated an average running speed based on total revolutions divided by the number of 1-min intervals with any revolutions [2], our approach does not require averaging over intervals. In fact, speeds can be determined for an individual rotation regardless of how quick or slow the animal rotates it (Fig. 1b). More importantly, however, this approach prevents averaging in time points with which the animal is either not moving or is no longer in the wheel (e.g. 1 rotation in 1-min).

Finally, wheel-skill learning is based on previous findings that noted that during the beginning of the running-wheel training (i.e. when naive rats are first exposed to the running wheel), rats are unable to run with an appropriate speed to remain at the bottom of the wheel. As a result, the rat often moves too fast or too slow relative to the speed of the wheel, thus generating a “wobble” [10]. While running-wheel training is a pre-requisite for wheel-skill learning [10], this has been difficult to illustrate quantitatively. However, by combining real-time video with a real-time continuous positional waveform (Fig. 3; Additional file 2: Video S1), running-wheel learning can also be objectively monitored and quantitatively illustrated over time.

### Conclusion

In short, we have developed a system for assessing wheel-running behaviour in rodents amenable to real-time integration with a high-speed video suitable for most typical wheel-running experiments. In addition, our system allows for detailed information like wheel rotation fluidity, directionality, instantaneous velocity, average and maximum velocity, acceleration, total number of rotations, and the temporal pattern of wheel-running behaviour to be derived from a single trace. While it is ideal for evaluating mechanisms of procedural motor learning using wheel-skill learning, it can be applied to many other experimental paradigms given that detailed information required for most wheel-running experiments can be derived from a single continuous trace.

## Methods

Male Sprague–Dawley rats (6 weeks old) were housed under standard laboratory conditions in the University of Calgary Animal Resource Center in accordance with our approved Animal Care Committee protocol (AC12-0239). Animals had free access to food (standard rodent chow) and water. Wheel-skill learning is based on wheel-running [10]. Training and testing were performed in the same room, and before the day of wheel-skill learning, all animals (n = 8; randomly divided into two groups) were first habituated to the test/training room and wheel by placing them in the wheel under the locked condition for about 1 h. In addition, during the day of testing, the rat was again first placed inside the locked wheel for an approximately 2-min habituation period. The wheel with the rat was then gently rotated as occurs naturally during wheel running such that the rat (with head up [10]) was at a 90° position (“rotation phase”). Upon release (“release phase”), the wheel swings back and forth until the rat stops the swinging by counterbalancing. Therefore, wheel-skill performance was measured by quantifying the total or cumulative rotational distance of the wheel during the release phase until the wheel typically failed to rotate greater than 3° for 1500 ms. Performance was evaluated before (pre) and after (post) the training period in which the running-wheel group had free access to wheel running, while the wheel was locked in the locked-wheel group (~40 min). The running wheels were approved by the University of Calgary Animal Care Committee (AC12-0239) and consisted of a rotating metal chamber with a wire mesh floor (Additional file 1: Figure S1; diameter, 36 cm; width, 14 cm) attached to a stationary metal wall with an access opening that could be closed. All data is presented as mean ± SEM and two-tailed t test p value determination was accomplished using the statistical software GraphPad Prism. Data for traces were acquired at 20 or 100 Hz. All attempts were made to minimise the handling of animals and the number of animals used.

## Availability of supporting data

The design of the system using LabVIEW system design software (National Instruments) supporting the results of this article are included within the article and its additional file.

## Additional files

10.1186/s13104-016-2059-6 A schematic overview of the system used for assessing wheel-running behaviour in rodents. A: A picture of the running wheel, angular encoder, and dimensions. B: A schematic overview of the system using LabVIEW system design software (National Instruments). A laptop computer (IBM ThinkPad) with a National Instruments A/D board (NI DAC-Card 6024E, 200 kSamples/s, 16 channels), a breakout box (National Instruments BNC), and a high speed IEEE 1394a port was used to run in-house software to collect raw position data.
10.1186/s13104-016-2059-6 Integration of waveform with high-speed video.
