# Supplementary material for: Development and testing of a new system for assessing wheel-running behaviour in rodents
Source: BMC Res Notes. 2016 May 5;9:262. doi: 10.1186/s13104-016-2059-6 (PMC4858930; doi:10.1186/s13104-016-2059-6)
Supplement: Supplementary file 1 — 10.1186/s13104-016-2059-6 A schematic overview of the system used for assessing wheel-running behaviour in rodents. A: A picture of the running wheel, angular encoder, and dimensions. B: A schematic overview of the system using LabVIEW system design software (National Instruments). A laptop computer (IBM ThinkPad) with a National Instruments A/D board (NI DAC-Card 6024E, 200 kSamples/s, 16 channels), a breakout box (National Instruments BNC), and a high speed IEEE 1394a port was used to run in-house software to collect raw position data. [file 13104_2016_2059_MOESM1_ESM.docx]

**Figure S1. A schematic overview of the system** **used for assessing wheel-running behaviour in rodents**

A: A picture of the running wheel, angular encoder, and dimensions. B: A schematic overview of the system using LabVIEW system design software (National Instruments). A laptop computer (IBM ThinkPad) with a National Instruments A/D board (NI DAC-Card 6024E, 200 kSamples/s, 16 channels), a breakout box (National Instruments BNC), and a high speed IEEE 1394a port was used to run in-house software to collect raw position data.
